# Supplementary material for: VUStruct: a compute pipeline for high throughput and personalized structural biology
Source: bioRxiv. 2025 Mar 26:2024.08.06.606224. Originally published 2024 Aug 7. Preprint. [Version 2] doi: 10.1101/2024.08.06.606224 (PMC11326201; doi:10.1101/2024.08.06.606224)
Supplement: Supplement 1 [file media-1.pdf]

Supplemental Information

Introduction

While VUStruct has broad application to most any set of VUSs, we specifically share here the workflow we have developed to support weekly clinical cases for the Vanderbilt UDN. VUStruct allows our Personal Structural Biology (PSB) team to inform the clinic from the intersection of structural biology, genetics, and computation.

As its final automated step, the pipeline creates a patient case website of detailed calculation outputs and interactive protein visualizations. Our lead structural biologist reviews this generated website and applies her experience in computational structural biology to evaluate and curate the detailed results in broader biochemical and structural context. She particularly considers confidence and quality metrics for individual calculations and structural models. At the weekly meeting, following clinician discussions of candidate genes, she quickly summarizes the curated VUstruct outputs as a single page multicolor spreadsheet. She presents that report in context of the meeting's critical preceding discussion.

Suggestions for Clinical Case Support

Our summary variant classifications support the discussions of the clinical team (1,2) and serve as inputs to their generation of hypotheses. In the best cases, our insights lead to testable biophysical/biochemical mechanistic explanations for pathogenicity. Uncertainties abound not only in the calculation algorithms, but also in their inputs. Given these uncertainties, the team curates raw calculation outputs through the lens of its structural biology experience. From hundred(s) of calculation details, we whittle our weekly end-product to a single page. Our manually edited case summary grid communicates, for each missense VUS (rows) and for each calculation (columns), our initial sense of whether a variant might impact function. We report our final recommendations in the right column with colors "no", "maybe", or "yes" for variants meriting further investigation.

Some calculations cannot be run due to a variety of issues ranging from lack of quality structural coverage for the variant, to insufficient available variants for PathProx spatial analyses, to variant-specific technical reasons. These missing cells are left blank in the summary. VUStruct creates this spreadsheet for each case, as an editable template. Supplemental Figure 1 is an edited summary spreadsheet from a recent case:

Gene	Uniprot ID	variant	ddG destabilizing	PathProx 3D hotspot w/ ClinVar	PathProx 3D hotspot w/COSMIC	PPI	AlphaMissense Score	PTM	Digenic interaction by DiGePred or DIEP	Worth pursuing?	side notes
AMOT	Q4VCS5-1	V472L	-0.5		0.1	0.9	0.9	Phosphotyrosine		PPI + PTM + AM	
CITED4	Q96RK1	A106T	9.0		0.1		0.07				Low confidence model
PAQR6	Q6TCH4-1	A230V	-1.1		0.2		0.64			ddG + AM	
NLRP1	Q9C000-1	S38P	-1.73 - 10.41	-0.3	0.0	0.6	0.06	Phosphothreonine		ddG + PPI + PTM	
ACADM	P11310-1	K329E	5.5	0.1	0.3	0.9	0.51		Diep w. UQCRC2, LPL, FARS	ddg + PPI + Digenic	variant is in oligomer interface
CBS	P35520-1	R369C	4.7	0.3	0.2		0.15	SUMOylation		ddG + Clinvar + PTM	dimer interface variant
CDC42BPA	Q5VT25-1	T419M	-1.8		0.1	0.8	0.08	Phosphoserine		ddG + PPI + PTM	
CNOT1	A5YKK6-1	R807G		0.0	0.0		0.23				
COX10	Q12887-1	C343R	1.4	0.4	0.2		0.18		Diep w. UQCRC2	ddG + Clinvar + Digenic	
CR1	P17927	R1564Q			0.0	0.5	0.09	Phosphoserine			
DIS3L2	Q8IYB7-1	A83V	7.5	0.3	0.5		0.95			ddG + AM	
DLL1	O00548-1	N300K	4.1		0.1		0.93	O-linked_glycosylation		ddG + AM + PTM	
FARSB	Q9NSD9-1	R98W	-2.9	0.5	0.1		0.2		Diep w. ACADM	ddG + Clinvar + Digenic	
HYOU1	Q9Y4L1-1	V239I	-0.32 - 5.44				0.08			ddG	
IRF8	Q02556	R170G	0.4	0.0	0.0	0.7	0.1			PPI	
LPL	P06858	N318S	-0.9	0.4	0.2		0.07		Diep w. MSR1, ACADM	Digenic + Clinvar	
MAPK8IP3	Q9UPT6-1	V12M	1.6	0.0	0.0	0.7	0.15			PPI	
PIK3CB	P42338	V199I	-1.7		0.0		0.08			ddG	ras binding domain variant
S1PR3	Q99500	M365L	-1.9		0.0		0.07			ddG	Low confident model
UQCRC2	P22695	T142I	2.7	0.6	0.1	0.9	0.31	Methylarginine	Diep w. ACADM, COX10	G + Clin. + PPI + PTM + Dige.	

legend
Yes
Maybe
No

Supplemental Figure 1. VUstruct calculation results are presented as a single case summary spreadsheet, which is manually edited from a VUStruct-generated template. The initial PSB team recommendations are shown in a “Worth Pursuing?” column and presented in context of preceding clinician discussions.

Our meeting preparations often include literature searches and give closer attention to experimentally determined structures. These insights can identify additional protein structural nuances and reveal possible variant impacts on dimerization surfaces or other interaction sites.

During the case review meeting, the PSB team lead listens closely to the case discussion and succinctly relays our findings in context. The lead doesn’t report on the entire spreadsheet. Rather, she provides additional details or perspective from our results that directly support or challenge the emerging hypotheses of causal genes. The team lead may also suggest ongoing consideration of additional genes, where our calculations strongly implicate them, so long as preceding discussion has not definitively eliminated them.

Questions from the team are always welcomed and taken offline when follow-up research is needed. The PSB team is always available for additional pipeline runs, and for supplementary modeling to dig deeper into specific variants and report back on current or prior cases. Indeed, the VUstruct pipeline’s integrated calculations are but a small sample of computational methods available to us.

VUstruct Details: Calculations and their Interpretation

The pipeline-launched calculations typically output numerical values which include biophysical energy change, pathogenicity predictions, or probability of location at functional protein sites. Along with these raw numbers, the calculations deliver critically important statistical significance intervals and metrics of predictive confidence.

We now describe the specific calculations in greater detail, along with the ever-evolving interpretational guidelines that we have developed to summarize results.

Rosetta $\Delta\Delta G_{folding}$

While proteins are tolerant of (i.e., are functional after) most single random amino acid substitutions(3,4). However, protein folding is an extraordinarily subtle phenomenon of nature. For globular protein, $\Delta G_{unfolded \text{-->} folded}$ measurements have long been understood to lie in the range [-5, -15] kCal/mol(5). Thus, the free energy of protein folding is comparable to the enthalpy of a couple of hydrogen bonds - remarkably small relative to the sum of energetics acting between all the atoms of a protein or the global loss of entropy on folding. From basic thermodynamics, it must follow that a 1.5 kCal/mol increase to $\Delta G_{folding}$ drives a 10-fold reduction in folded protein concentration and calculations have suggested that 20% of deleterious missense variants cross this low energetic barrier(6). Though the prevailing theoretical intuition around protein folding thermodynamics is arguably oversimplistic when considering more complex proteins and *in-vivo* chaperones (7), the energetic impact of a single amino acid variant can obviously be sufficient to disrupt folding. Trivial mechanistic explanations might include disruption of a critical hydrogen bond, loss of a disulfide bride, a new steric clash – any of which has the *potential* to render a protein unstable and disrupt function. Importantly, our calculations do not claim to calculate the thermodynamics of ΔG. Rather, our alchemistic hope is that the comparison of varying energetics before and after variation will be sufficient to estimate a *change* in ΔG, a $\Delta(\Delta G_{unfolded \text{-->} folded})_{variant}$.

In comparison to the subtlety of nature, our Rosetta ΔΔG Cartesian(8,9) calculations are a fast approximation. From a background total computed energy which includes hundreds or thousands of amino acid atomic interactions, (easily summing to 100,000s of kcal/mol) the Rosetta calculation attempts to determine how the new variant side chain would fit in a still-folded protein, allowing for limited rearrangement of nearby amino acids. The approximation of general protein rigidity, the treatment of only protein monomers without bound ligands, and the lack of membrane context make for a convenient and fast-running calculation with a result that we do not trust blindly. Indeed, detailed benchmarking of the computed ΔΔGs on "simple" soluble monomeric proteins found its performance only somewhat helpful in the task of engineering large libraries of protein designs, an application quite distanced from characterizing individual protein variants in individualized patients(9).

We nonetheless find the ΔΔG calculation illuminating in a *reverse* sense. When variants have a small calculated energetic impact, in the interval [-2,2] kCal/mol for our purposes, we will generally classify these as benign, depending on structural context. Outside that small range, we look more closely at 3D structures interactively, and we admit to layering subjectivity on the interpretation and classification of these results. We discount the predictive power of the calculation when it involves residues from low-confidence models, or poorly resolved experimental structure. Often, we find that the ΔΔG calculation will simply highlight amino acid changes that are predictably deleterious without any detailed calculation (ex: to or from proline or glycine, introduction of tryptophan, changes from large to small, hydrophobicity, etc.). In the end, red/yellow/green are assigned to the spreadsheet cell after some reflection. More robust analysis techniques are available if there is interest from the clinical team.

PathProx: Pathogenic Proximity

PathProx(10,11) analyzes the placement of Clinvar(12), Gnomad(13), and random (null hypothesis) genetic variants on pipeline-selected 3D structures, and calculates whether the location of patient's VUS better clusters with the pathogenic or benign variant sets. Citation (10) offers a thorough explanation of the algorithm's mathematics and machine-learning approach. The intuitive rationale for the algorithm comes from the observation that, often, when we map variants onto amino acid side chains, we observe a higher

percentage of "known pathogenic" variants clustering inside smaller spheres vs. randomly distributed variants. This technique identifies 3D hot-spots in the cartesian space of the folded protein.

For each transcript PathProx mines the Clinvar database for variants annotated as "likely pathogenic" or "pathogenic." Benign variants are harvested from the Genome Aggregation Database gnomAD and filtered for MAF > $1x10^{-5}$. Roughly, we only accept as benign those variants which are found in at least 2 gnomAD sequences, a threshold that well contrasts to the rarity of UDN patient variants.

As a control, the PathProx algorithm computes a null distribution via thousands of random distributions of both N_{benign} and $N_{pathogenic}$ spheres on the then asks: "Relative to random positioning of N spheres, how do these specific N pathogenic (or benign) variants seem to cluster?". Often, we see significantly tighter clustering of the known pathogenic variants than would be expected from our random sampling. Simultaneously we see more diffuse clustering of benign variants vs. random placements. When we have both observations, and when our VUS fits more closely with the tighter clustering pathogenic set, we identify it as pathogenic, and look more closely at the 3D context of the variant sets.

PathProx cannot always make a prediction and for these cases we leave a blank cell in our summary spreadsheet. PathProx minimally requires 3 pathogenic variants, and often leave-one-out validation tells us (via low AUC), that the predictive power of the algorithm is not strong. When the calculation metrics imply confidence, we add a "no", "maybe", or "yes" to the PathProx-Clinvar column of the final spreadsheet.

As with the ΔΔG calculations, the PathProx algorithm is perhaps best validated across large protein datasets(10). Nonetheless, a strong case has been made for PathProx's power to explain disease in individual patients(11).

As with ΔΔG, the PathProx algorithm was benchmarked on protein monomers. We nonetheless run it also on multimers from Swiss-model and the PDB. We cautiously consider these multimeric calculations in our analysis – as homomeric structures introduce symmetries of variant placements that the algorithm may not be adept at scoring.

Digenic Disease Prediction

Disease emergence through compound heterozygous variation is well understood. Individually, a Proband's disease-free parents' gene functions enough under single variation. But the combination of gene variants from each parent triggers creates the observed phenotype in the proband. Thus, compound heterozygous variant pairs are always given special attention in each week's UDN review.

Multigenic disease can analogously arise when two genes in a single pathway are damaged via variations inherited from each parent.

An increasing database of annotated digenic(14) and multigenic(15) diseases is being curated from the literature. DiGePred(16) was an initial exploration in our lab, as to whether latent digenic diseases might be predicted through machine learning strategies. Another group broadened the training inputs, while retaining a similar machine architecture, to create DIEP(17)

These algorithms are both challenged by the relatively small training data sets. DIEP has higher sensitivity, at a cost of more frequent false positive predictions. We find DiGePred is perhaps too conservative in contrast, achieving a false positive rate of 0.1% vs. 4.4% for DIEP. DiGePred is noteworthy in that it retains

literature references, and outputs in an interactive JavaScript app where we can explore thresholds of confidence. We look forward to developing and integrating further developments in di- and multi-genic disease prediction.

Protein-Protein Interaction (PPI) Site Prediction

Many proteins function through binding to other protein partners, and approximately 60% of disease-associated missense mutations have been noted to perturb PPIs(18). Amino acid variants in interaction surfaces could have negligible impact in folding energetics and still be deleterious through disruption of protein binding to usual partners. The ScanNet(19) machine learning algorithm was trained through analysis of the Dockground(20) database of 3D protein-protein interacting structures. Operationally, for each variant position covered by an Alphafold model, we ask ScanNet to predict whether the position participates in a PPI binding surface. We report any variant position to which ScanNet assigns at least 50% probability of being involved in a PPI.

PTM

Post-translational modifications to proteins are often critical to function. However, these modifications (phosphorylation, glycosylation, and others) are typically unresolved in experimental X-ray structures, and never seen in model structures. Sequence-based prediction of post-translational modification sites has been evolving in recent decades, and we have integrated MutsiteDeep(21), the first deep-learning framework for prediction of these sites. We are particularly interested in potentially impacted PTMs in the vicinity of a VUS, and we report VUSs at sites predicted to have both at least a 50% likelihood of post-translational modification and fall within an approximate 8Å sphere around the VUS.

CONSURF and COSMIS

Residue positions which vary infrequently across species exhibit evolutionary conservation and this is already an input to the clinic via GERP(22) and GERP++(23) scores.

As structural biologists, we are interested not only in conservation for specific amino acids in a sequence, but also in the conservation, and visualization, of the surrounding 3D protein context. ConSurf(24) colors every residue based on rates of evolution from phylogenetic tree analysis (25).

The Contact Set MISsense tolerance algorithm, COSMIS (26) was developed in our lab. In contrast to ConSurf, which quantifies evolutionary constraint based on analysis of sequence alignments across diverse species, COSMIS quantifies evolutionary constraint over more recent evolution by analyzing patterns of genetic variation across diverse human populations. It also leverages the 3D spatial context of proteins to better estimate the evolutionary constraint on protein positions of interest. Supplemental Figure 2, below, is an example depiction of COSMIS scores.

Summary

By integrating a wide range of 3D structural calculations for each weekly UDN case, VUStruct has proven itself invaluable to the PSB team as it informs the clinic towards generation of hypotheses that explain variant pathogenicity.

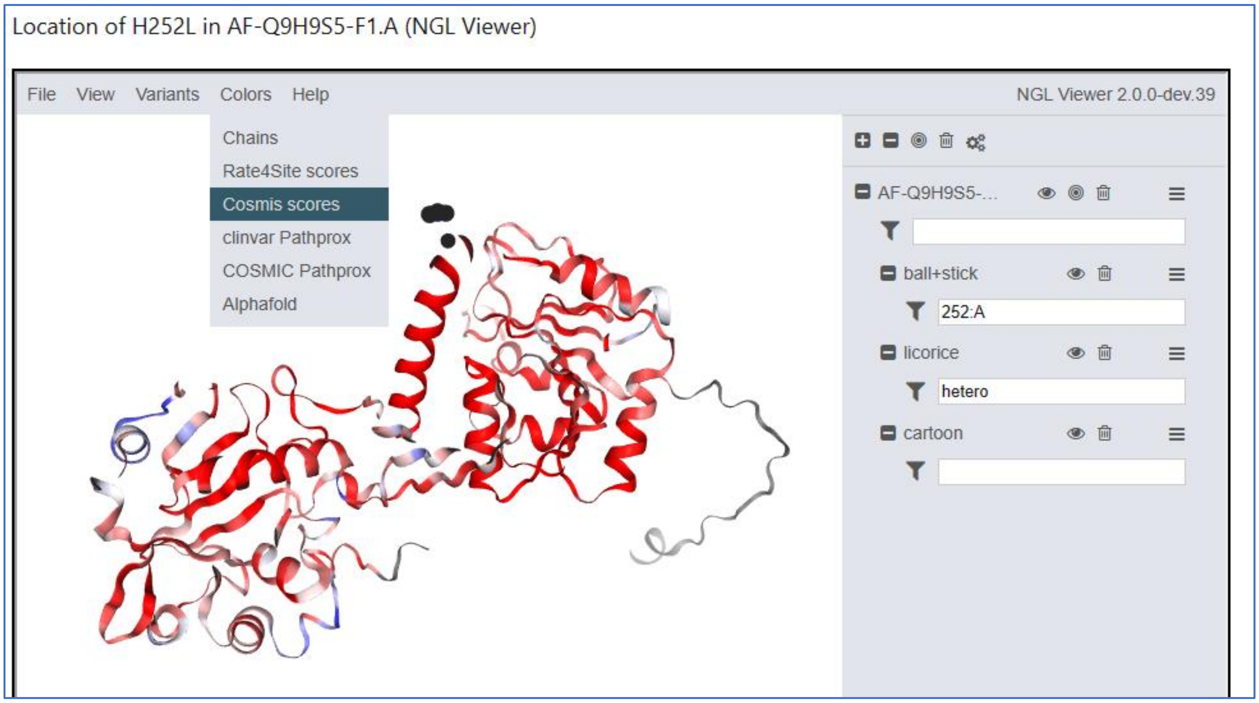

Supplemental Figure 2. COSMIS scores are depicted on the protein backbone. Red communicates high conservation. Blue tolerance to variation. This coloring scheme is also available for scores from CONSURF and PathProx. The colors in the example above reflect a general observation, that residues in highly structured protein regions tend to be more conserved across sequences, whether compared intra- or inter-species. Our pipeline additionally can rainbow-color chains in complex structures and show Alphafold pLDDTs with standard colors for those confidence metrics.

References

1. MacArthur DG, Manolio TA, Dimmock DP, Rehm HL, Shendure J, Abecasis GR, et al. Guidelines for investigating causality of sequence variants in human disease. Nature. 2014 Apr 23;508(7497):469–76.

2. Richards S, Aziz N, Bale S, Bick D, Das S, Gastier-Foster J, et al. Standards and guidelines for the interpretation of sequence variants: a joint consensus recommendation of the American College of Medical Genetics and Genomics and the Association for Molecular Pathology. Genetics in Medicine. 2015 May;17(5):405–24.

3. Guo HH, Choe J, Loeb LA. Protein tolerance to random amino acid change. Proceedings of the National Academy of Sciences. 2004 Jun 22;101(25):9205–10.

4. Bowie JU, Reidhaar-Olson JF, Lim WA, Sauer RT. Deciphering the Message in Protein Sequences: Tolerance to Amino Acid Substitutions. Science (1979). 1990 Mar 16;247(4948):1306–10.

5. Pace CN. Conformational stability of globular proteins. Trends Biochem Sci. 1990 Jan;15(1):14–7.

6. Berliner N, Teyra J, Çolak R, Garcia Lopez S, Kim PM. Combining Structural Modeling with Ensemble Machine Learning to Accurately Predict Protein Fold Stability and Binding Affinity Effects upon Mutation. PLoS One. 2014 Sep 22;9(9):e107353.

7. Sorokina I, Mushegian AR, Koonin E V. Is Protein Folding a Thermodynamically Unfavorable, Active, Energy-Dependent Process? Int J Mol Sci. 2022 Jan 4;23(1):521.

8. Park H, Bradley P, Greisen P, Liu Y, Mulligan VK, Kim DE, et al. Simultaneous Optimization of Biomolecular Energy Functions on Features from Small Molecules and Macromolecules. J Chem Theory Comput. 2016 Dec 13;12(12):6201–12.

9. Frenz B, Lewis SM, King I, DiMaio F, Park H, Song Y. Prediction of Protein Mutational Free Energy: Benchmark and Sampling Improvements Increase Classification Accuracy. Front Bioeng Biotechnol. 2020 Oct 8;8.

10. Sivley RM, Dou X, Meiler J, Bush WS, Capra JA. Comprehensive Analysis of Constraint on the Spatial Distribution of Missense Variants in Human Protein Structures. The American Journal of Human Genetics. 2018 Mar;102(3):415–26.

11. Sivley RM, Sheehan JH, Kropski JA, Cogan J, Blackwell TS, Phillips JA, et al. Three-dimensional spatial analysis of missense variants in RTEL1 identifies pathogenic variants in patients with Familial Interstitial Pneumonia. BMC Bioinformatics. 2018 Dec 23;19(1):18.

12. Landrum MJ, Lee JM, Benson M, Brown GR, Chao C, Chitipiralla S, et al. ClinVar: improving access to variant interpretations and supporting evidence. Nucleic Acids Res. 2018 Jan 4;46(D1):D1062–7.

13. Karczewski KJ, Francioli LC, Tiao G, Cummings BB, Alföldi J, Wang Q, et al. The mutational constraint spectrum quantified from variation in 141,456 humans. Nature. 2020 May 28;581(7809):434–43.

14. Gazzo AM, Daneels D, Cilia E, Bonduelle M, Abramowicz M, Van Dooren S, et al. DIDA: A curated and annotated digenic diseases database. Nucleic Acids Res. 2016 Jan 4;44(D1):D900–7.

15. Nachtegael C, Gravel B, Dillen A, Smits G, Nowé A, Papadimitriou S, et al. Scaling up oligogenic diseases research with OLIDA: the Oligogenic Diseases Database. Database. 2022 Apr 12;2022.

16. Mukherjee S, Cogan JD, Newman JH, Phillips JA, Hamid R, Meiler J, et al. Identifying digenic disease genes via machine learning in the Undiagnosed Diseases Network. The American Journal of Human Genetics. 2021 Oct;108(10):1946–63.

17. Yuan Y, Zhang L, Long Q, Jiang H, Li M. An accurate prediction model of digenic interaction for estimating pathogenic gene pairs of human diseases. Comput Struct Biotechnol J. 2022;20:3639–52.

18. Sahni N, Yi S, Taipale M, Fuxman Bass JI, Coulombe-Huntington J, Yang F, et al. Widespread Macromolecular Interaction Perturbations in Human Genetic Disorders. Cell. 2015 Apr;161(3):647–60.

19. Tubiana J, Schneidman-Duhovny D, Wolfson HJ. ScanNet: an interpretable geometric deep learning model for structure-based protein binding site prediction. Nat Methods. 2022 Jun 30;19(6):730–9.

20. Kundrotas PJ, Kotthoff I, Choi SW, Copeland MM, Vakser IA. Dockground Tool for Development and Benchmarking of Protein Docking Procedures. In 2020. p. 289–300.

21. Wang D, Zeng S, Xu C, Qiu W, Liang Y, Joshi T, et al. MusiteDeep: a deep-learning framework for general and kinase-specific phosphorylation site prediction. Bioinformatics. 2017 Dec 15;33(24):3909–16.

22. Cooper GM, Stone EA, Asimenos G, Green ED, Batzoglou S, Sidow A. Distribution and intensity of constraint in mammalian genomic sequence. Genome Res. 2005 Jul;15(7):901–13.

23. Davydov E V., Goode DL, Sirota M, Cooper GM, Sidow A, Batzoglou S. Identifying a High Fraction of the Human Genome to be under Selective Constraint Using GERP++. PLoS Comput Biol. 2010 Dec 2;6(12):e1001025.

24. Ashkenazy H, Abadi S, Martz E, Chay O, Mayrose I, Pupko T, et al. ConSurf 2016: an improved methodology to estimate and visualize evolutionary conservation in macromolecules. Nucleic Acids Res. 2016 Jul 8;44(W1):W344–50.

25. Pupko T, Bell RE, Mayrose I, Glaser F, Ben-Tal N. Rate4Site: an algorithmic tool for the identification of functional regions in proteins by surface mapping of evolutionary determinants within their homologues. Bioinformatics. 2002 Jul 1;18(suppl_1):S71–7.

26. Li B, Roden DM, Capra JA. The 3D mutational constraint on amino acid sites in the human proteome. Nat Commun. 2022 Jun 7;13(1):3273.
